# Leveraging Digital Media to Promote Youth Mental Health: Flipping the Script on Social Media-Related Risk

**DOI:** 10.1007/s40501-024-00315-y

**Published:** 2024-04-08

**Authors:** Jessica L. Hamilton, John Torous, Hannah S. Szlyk, Candice Biernesser, Kaylee P. Kruzan, Michaeline Jensen, Jazmin Reyes-Portillo, Brian A. Primack, Jamie Zelazny, Paul Weigle

**Affiliations:** 1 Rutgers University, New Brunswick, NJ, USA; 2 Harvard Medical School, Boston, MA, USA; 3 Washington University in St Louis, St Louis, MO, USA; 4 University of Pittsburgh, Pittsburgh, PA, USA; 5 Northwestern University, Chicago, IL, USA; 6 University of North Carolina-Greensboro, Greensboro, NC, USA; 7 Montclair State University, Montclair, NJ, USA; 8 Oregon State University, Corvallis, OR, USA; 9 University of Connecticut School of Medicine, Farmington, CT, USA; 10 Department of Psychology, Rutgers University, 53 Avenue E, Piscataway, NJ 08854, USA

**Keywords:** Digital media, Social media, Adolescence, Mental health, Prevention, Intervention

## Abstract

**Purpose of Review:**

Despite growing public concern about the negative impact of digital media for mental health problems, there are key ways in which digital media can be leveraged to prevent such outcomes. This article reviews research exploring the ways that digital media, particularly social media, can be used to prevent negative mental health outcomes and promote youth mental health and well-being.

**Recent findings:**

Research indicates that media can be protective against mental health problems and promote mental health by enabling social support and destigmatizing mental illness, especially for youth with limited resources. Media also can be leveraged to identify those at risk, to educate, provide resources, and promote well-being, and to track symptoms and intervene to prevent or mitigate negative mental health outcomes. There is limited research on interventions designed to reduce the negative effects of digital media on mental health, especially those that harness media itself, a critical area of future research.

**Summary:**

This article provides a summary of the current evidence on this topic, highlights key directions for future research, and provides evidence-based recommendations for adolescents, families, educators, clinicians, industry, and policy-makers to prevent mental health problems related to media.

## Introduction

Considerable research has examined the potential links between digital media, particularly social media, and youth mental health [1••]. While digital media elevates risk for mental health problems for some youth, it can also be leveraged to prevent negative mental health outcomes related to its use and promote well-being. In this review, we highlight three ways in which digital media can promote youth mental health by (1) facilitating and strengthening social connection, particularly for vulnerable youth; (2) identifying risk for mental health problems and disseminating mental health education and resources; and (3) digital interventions designed to improve mental health related to media use. This article briefly reviews the current state of research on these topics and identifies current limitations and key directions for future research. Based on the current state of the field, recommendations are provided for youth and their families, clinicians and educators, and policy-makers and industry.

## Digital Media to Promote Social Connection and Support to Protect Against Mental Health Problems

To date, most research has focused on the role of social media to facilitate social connections and relationships [2]. Social support and close relationships are critical during the developmental period of adolescence and can protect against mental health problems [3]. For instance, social media can facilitate self-disclosure, thereby strengthening offline relationships through increased intimacy [4]. The opportunity to connect with people who share beliefs, values, experiences, and/or identities can enable a sense of belonging and community [5], which may be particularly important for youth with existing physical or mental health problems [6]. Social media platforms can help adolescents cope with difficult times through support from others [7], including as a source of validation, social connection, and connection with others who have similar diagnoses [8]. Consistent with this, a recent survey found 35% of teens believe social media is mostly beneficial, citing social connection as one of its core benefits and that it facilitates support during trying times [9]. Based on the research to date, guidelines and recommendations support the use of social media in a way to “create opportunities for social support, online companionship, and emotional intimacy that can promote healthy socialization” [10••].

Youths with fewer family, school, and community resources and greater stressors may be more likely to benefit from the protective aspects of media use. Individuals who hold minoritized identities (e.g., racial, ethnic, gender, sexual, religious) may especially benefit from protective aspects of media, such as through connection with online communities who share their identities [11•]. For LGBTQIA + individuals, especially those lacking acceptance at home or school, online communities provide an important space to find acceptance and connection with peers who share experiences [12] or buffer against stigma and minority stress [13]. Young people with minoritized racial-ethnic identities likewise can experience online spaces as venues for identity exploration, empowerment, civic engagement, connection with peers, and building social capital [14, 15]. Examination of within-group experiences on social media is key to understanding who is most likely to benefit and what factors (e.g., racial-ethnic identity, community support) may buffer against its negative effects on mental health [16].

## Digital Media to Identify Risk and Disseminate Mental Health Education and Resources

Social media holds promise as a means to identify mental health risk, both at an individual level (e.g., via distress posts) and as an indication of broader patterns of risk (e.g., via online mental health dialogue). For example, Twitter and Instagram references to the show “13 Reasons Why” were linked to increased youth suicide deaths during the same time period [17–19]. Conversely, social media references to American rapper Logic’s song “1–800–273–8255” (the phone number of the National Suicide Prevention Lifeline) were linked to increased help-seeking behaviors related to suicidality [20, 21]. Novel research seeks to develop tools to extract and analyze the content of youth smartphone engagement, such as text posted to social media, exchanged in private communications, or searched via web browser, to predict and prevent health risk behaviors like suicide. This information can then inform the development of just-in-time interventions, although interventions based on such evidence bear potential pitfalls including ethical concerns [22].

Media also can be leveraged to destigmatize mental illness and related help-seeking. A study of Twitter posts connected to World Mental Health Day found that the majority were inspirational or offered advice for thriving despite mental illness [23]. One review determined that social media can be an effective tool to increase awareness about mental health, highlighting its potential to disseminate mental health education [24]. Adolescents primarily seek and access health information online, including about mental health [25], which is typically considered beneficial by teens [26•]. Adolescents also feel more comfortable disclosing sensitive personal information anonymously online compared to an in-person or non-anonymous format [27], which may promote help-seeking behaviors. Among adolescents with mental health concerns, 90% use the internet to access-related information [28]. Accessing accurate mental health information through social media can help youth learn about mental health problems, identify when to seek help, and access needed resources. Some platforms (e.g., TikTok) are perceived by adolescents as more likely to have misinformation or negative mental health information than others (e.g., YouTube) [26•], which may be an important consideration for mental health professionals using media to deliver content.

## Digital Media As a Tool for Mental Health Intervention

Digital media holds great promise as a scalable, accessible, and low-cost tool for the prevention and treatment of mental health problems [29•]. Digital mental health interventions (DMHIs) may be particularly beneficial for reaching adolescent populations with medical comorbidities [30] or disordered eating [31]. These tools are generally acceptable to youth [32] and can more easily fit within their daily lives and media use patterns. Further, DMHIs can further support underserved and minoritized populations who face barriers to accessing appropriate mental health care [33]. Steps to address the increasing digital divide are important to ensure broader access. Prior scoping and systematic reviews found preliminary evidence supporting effectiveness of using video games [34] and social media-based interventions [35••] to improve mental health. For instance, Kruzan and colleagues [32] concluded that social media interventions that include psychoeducation, leverage peer support, and faciliate sharing appear feasible, acceptable, and possibly efficacious for young people with mental health symptoms, though more research is needed. Digital tools also can be used to monitor symptoms and treatment progress, including tracking the mental health effects of media habits and experiences.

Despite concerns about negative mental health effects of social media and digital media use, there is limited research evaluating interventions, particularly digital interventions, designed to promote healthy media use. Most research on this topic has focused on interventions to reduce problematic video gaming or internet use, with recent randomized controlled trials suggesting their effectiveness [36, 37]. For example, a manualized cognitive behavioral therapy–based preventive group intervention significantly reduced symptom severity of gaming disorder or unspecified internet use disorder among adolescents [36]. A small randomized controlled trial found that adolescents who engaged in daily monitoring during a brief abstinence from gaming improved their attitude toward gaming [37].

The limited empirical interventions concerning social media focus mostly on abstinence or brief breaks from media among young adults, with some studies suggesting that breaks from social media may improve mental health symptoms [38]. Several emerging interventions use mindfulness [39] and values-alignment [40] to self-regulate social media use, and limit excessive scrolling and unwanted use. Some studies in young adults suggest that instilling a sense of agency over social media may result in more intentional and moderate use, but only for individuals who engaged in higher-level reflection [41]. Fewer interventions have leveraged digital media to promote media use that is beneficial to mental health, though work is underway. One exception is the online suicide prevention tool #chatSafe, which is a media guide designed to help teens communicate safely online about suicide [42]. Initial results suggest it helped individuals identify and support others at risk for suicide [43].

## Limitations and Summary

Social media can prevent negative mental health outcomes through its protective components, such as social support, particularly for youth lacking offline support. Digital media also can be used to promote mental health, including as a tool for education, prevention, or intervention. Research investigating the effectiveness of various methods of using media to improve mental health is still in its infancy, particularly related to promoting healthy media use among adolescents.

## Directions for Future Research

1. How to harness media to support youth with mental health problems? There is a need to better understand how to leverage the beneficial components of media to promote mental health. For instance, teaching teens how to care for themselves and support others (e.g., similar to #chatsafe) at a larger scale and in different contexts can promote healthy media use and prevent negative mental health outcomes.
2. Do media-driven risk identification (i.e., flagging) and intervention messages (e.g., violence, suicide, domestic violence) effectively mitigate negative mental health outcomes? [44] Preliminary research on using media screening for eating disorders among teens shows promise in facilitating needed treatment and support, though more work is needed to understand the effectiveness of this approach for identifying those at risk [45].
3. Can we develop effective, large-scale interventions using digital media to reduce negative mental health outcomes related to media and promote well-being? There is a critical need for scalable, accessible, and developmentally appropriate digital interventions to prevent and treat adolescent mental health problems related to media. There is a need to develop validated screening and assessment tools that may be employed in clinical and educational settings to identify and characterize youth most at risk for social media’s negative effects. Further research is needed on how digital tools can help adolescents cope and self-regulate their media use, such as through *just-in-time* interventions.

Based on the current state of research, the following overall recommendations are provided, including specific recommendations across different key informants:

1) Make social media education and digital literacy programs accessible for teens, parents, clinicians, and educators to promote healthy use.

- Educators and schools: Provide evidence-based educational materials concerning media and mental health, as well as programs to treat problematic use in youth of different age ranges and identities. Teach critical evaluation of online information and recognition of unhealthy media habits and interactions via integration into lesson plans and socio-emotional learning programs.
- Caregivers and families: Consider family media use patterns to promote adaptive use among children. For instance, parents/caregivers can make use of the Family Media Use Plan developed by the American Academy of Pediatrics to open up communication about media use in the home and help parents and teens develop collaborative media rules. It’s also important for parents to consider their own media use behavior as elevated use among parents can negatively impact the parent–child relationship [46] (see here: https://www.healthychildren.org/English/fmp/Pages/MediaPlan.aspx)
- Researchers: Create digestible, acceptable, and accessible translational materials (e.g., blogs and social media content incorporating graphics), in partnership with key informants, to aid parents, teens, and clinicians identify risks and benefits of media related to mental health.

2) All key informants: Be intentional regarding use of teens’ social media data for prevention or intervention purposes to safeguard adolescent autonomy, privacy, and collaboration. While some social media data is public or accessed via school devices, all adults working with teens (e.g., clinicians, parents, schools, and researchers) should be respectful of adolescents’ privacy and confidentiality when retrieving and using this information, especially as it pertains to mental health. Retrieval for purposes of ensuring immediate safety (e.g., positing of suicide plan) is an important exception. Researchers and clinicians should be transparent with teen participants or clients about intentions to use their social media data in planning studies, interventions, and treatment, to ensure teen agency in the process.

3) Researchers and industry must collaborate to identify risk and improve how media affects the mental health of teens.

- Media industry: Media companies should grant greater access to social media data to health-focused researchers [47], as large-scale, timely data may answer key questions about which young people are most at risk and when. Twitter’s policy change in 2023 to restrict public access to its data suggests a concerning trend and highlights the urgency of collaborations between researchers and industry key informants. It also is important to improve access to data regarding social media algorithms, privacy, and health-related use of data (e.g., when data is being sent to law enforcement).
- Media industry/clinicians: Media companies should collaborate with clinicians to disseminate narratives concerning mental health that increase awareness, destigmatize, educate, and promote help-seeking behaviors, promoting positive outcomes.
- Media industry/researchers: Partnerships are critical to create promising preventative interventions built within existing social media platforms [48]. Researchers must be consultants and key informants to inform media development to minimize its negative mental health effects and maximize benefits [49].
- Government: Increase oversight regarding what content is permitted in online marketing (similar to restrictions on advertising of vapes), what data is collected, and how collected data is used (e.g., sale to marketing firms) [50]. Engage scientists and subject-matter experts in the development and implementation of policy around healthy media use to ensure evidence-based policies and guidelines that move beyond solely limiting access to social media.
